# First-principles study of AlGaSi_2_X_6_ (X = S, Se, Te) monolayers: structural, electronic, transport and photocatalytic properties

**DOI:** 10.1039/d6ra00973e

**Published:** 2026-05-06

**Authors:** Thi H. Ho, Tuan V. Vu, A. I. Kartamyshev, Dat D. Vo, Duy Khanh Nguyen, Nguyen N. Hieu

**Affiliations:** a Laboratory for Computational Physics, Institute for Computational Science and Artificial Intelligence, Van Lang University Ho Chi Minh City Vietnam thi.hohuynh@vlu.edu.vn tuan.vu@vlu.edu.vn; b Faculty of Mechanical, Electrical, and Computer Engineering, Van Lang School of Technology, Van Lang University Ho Chi Minh City Vietnam; c Institute of Research and Development, Duy Tan University Da Nang 550000 Vietnam; d Faculty of Natural Sciences, Duy Tan University Da Nang 550000 Vietnam

## Abstract

Density functional theory (DFT) calculations were employed to investigate quaternary AlGaSi_2_X_6_ (X = S, Se, Te) monolayers as two-dimensional semiconductors for photocatalytic and nanoelectronic applications. All three monolayers are predicted to be both dynamically and thermally stable and exhibit indirect band gaps that systematically decrease with increasing chalcogen atomic weight, from 2.79 eV for AlGaSi_2_S_6_ to 2.32 eV for AlGaSi_2_Se_6_ and 1.12 eV for AlGaSi_2_Te_6_. Vacuum-referenced band-edge alignments indicate that AlGaSi_2_S_6_ and AlGaSi_2_Se_6_ can thermodynamically drive overall water splitting under illumination, whereas AlGaSi_2_Te_6_ possesses an insufficient band gap to provide the required photovoltage. Gibbs free-energy profiles further support photoassisted hydrogen and oxygen evolution reactions (HER/OER) on the S- and Se-based monolayers. Moreover, AM1.5G solar spectrum estimates yield solar-to-hydrogen (STH) efficiencies of 3.90% for AlGaSi_2_S_6_ and 10.86% for AlGaSi_2_Se_6_. Deformation-potential analysis predicts electron-dominated transport with carrier mobilities reaching 1.7 × 10^3^ cm^2^ V^−1^ s^−1^, identifying AlGaSi_2_Se_6_ as the most promising overall candidate among these monolayers.

## Abstract 2

Density functional theory (DFT) calculations were employed to investigate quaternary AlGaSi_2_X_6_ (X = S, Se, Te) monolayers as two-dimensional semiconductors for photocatalytic and nanoelectronic applications.

## Graphical abstract

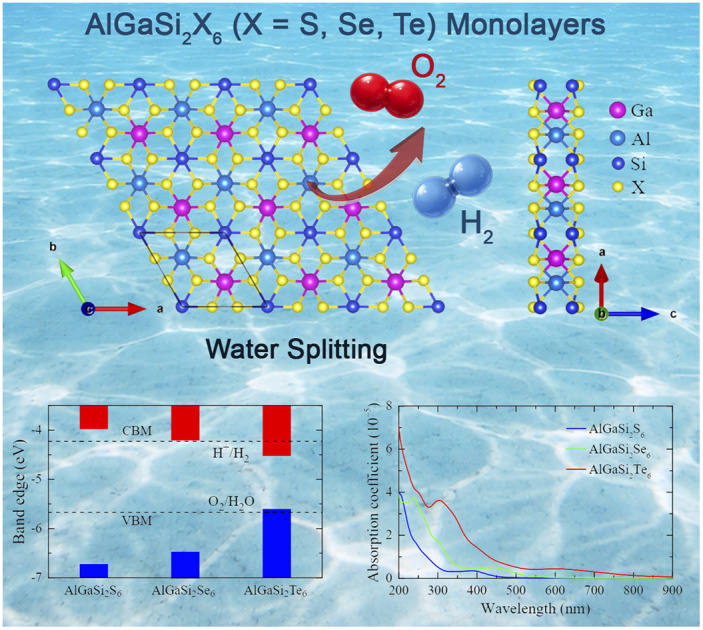

## 1 Introduction

The growing demand for clean and sustainable energy has intensified interest in photocatalytic overall water splitting as a direct route to solar hydrogen production.^1–3^ However, conventional semiconductor photocatalysts, such as TiO_2_, ZnO or bulk chalcogenides, often face intrinsic limitations, including wide band gaps, weak visible-light utilization, and fast electron–hole recombination, that collectively suppress solar-to-hydrogen efficiency.^4–6^ In this context, two-dimensional (2D) van der Waals monolayers provide a compelling platform because their atomically thin geometry maximizes exposed surface area and shortens the transport distance for photogenerated carriers, while quantum confinement and reduced dielectric screening enable wide tunability of band structure and optical response through composition, strain, and stacking/heterostructure engineering.^7–9^ For photocatalytic water splitting, an effective monolayer photocatalyst must simultaneously satisfy a suitable band gap and strong solar absorption, band-edge positions that can thermodynamically drive the H^+^/H_2_ and O_2_/H_2_O half-reactions, and efficient charge separation with favorable interfacial reaction kinetics to suppress recombination and overcome overpotentials.^10,11^ Accordingly, 2D chalcogenides and related engineered 2D systems have been widely explored as photocatalysts and photoelectrodes, where rational design, including defect/edge activation, surface functionalization, and van der Waals heterostructure construction, can jointly tune adsorption energetics, interfacial band alignment and internal fields at junctions, and carrier dynamics.^9,12–14^

Within the broad landscape of 2D candidates, non-TMD III–VI chalcogenide monolayers also offer an attractive and comparatively underexplored materials space for photocatalysis. For example, GaS, GaSe, GaTe and related III–VI compounds have attracted sustained attention because they combine tunable band gaps with favorable carrier and optoelectronic properties in the monolayer limit.^15^ Building on this platform, mixed-chalcogen Ga-based derivatives Ga_2_X_1_X_2_ (X_1_, X_2_ = S, Se, Te) provide an additional compositional degree of freedom, where they can be conceptually obtained by replacing one chalcogen sublayer in GaX with a different chalcogen while preserving a single-phase 2D lattice, enabling systematic tuning of band edges, work function, and surface chemistry.^16^ Consistent with this idea, recent first-principles studies on compositionally engineered III–VI systems of the general forms X_2_SSe, GaInX_2_, and InGaXY have shown that combining different group-III cations with mixed chalcogen terminations can simultaneously tailor the band gap, carrier effective masses, and interfacial charge-transfer characteristics, and that defect engineering may further optimize HER/OER energetics toward values attainable under illumination.^17–21^ Moreover, high-throughput screening efforts across chemically diverse 2D crystals have highlighted many candidates with improved predicted solar-to-hydrogen performance compared with conventional photocatalysts, underscoring composition engineering as a general design principle for 2D energy materials.^22–24^ Despite these advances, multiatomic III–IV–VI chalcogenide families that integrate light group-III cations (Al, Ga) with group-IV elements (Si) remain comparatively underexplored, particularly within quaternary A_2_B_2_X_3_Y_3_.

Herein, we propose and systematically investigate a new series of multiatomic monolayers AlGaSi_2_X_6_ (X = S, Se, Te) as promising 2D semiconductors for photocatalytic and nanoelectronic applications. Using first-principles calculations, we examine their structural, dynamical, mechanical and thermal stability as well as elucidate their electronic structures. In addition, we evaluate their transport properties, optical response and absolute band-edge positions to assess visible-light absorption and the capability for overall water splitting. Our results show that the AlGaSi_2_X_6_ monolayers enrich the growing family of 2D materials by offering a flexible platform where composition and chalcogen asymmetry can be used to optimize the balance between band gap, carrier mobility and intrinsic electric field, thus opening new opportunities for high-performance photocatalysts and multifunctional 2D devices.

## 2 Computational details

All first-principles calculations were performed within density functional theory (DFT) using the Vienna *Ab initio* Simulation Package (VASP).^25–27^ Core-valence interactions were described by the projector augmented-wave (PAW) method.^28,29^ Structural relaxations and electronic properties were obtained using the Perdew–Burke–Ernzerhof (PBE) exchange-correlation functional^30^ within the generalized gradient approximation (GGA), while the screened hybrid functional HSE06 (ref. 31) was employed to refine the band-gap values. In the HSE06 calculations, the standard VASP settings were used, namely an exact-exchange mixing fraction of AEXX = 0.25 and a screening parameter of HFSCREEN = 0.2 Å^−1^, without further tuning. Since the calculated hybrid-functional band gap is known to be sensitive to the exact-exchange mixing parameter, these settings are stated explicitly for reproducibility.^32^ Long-range dispersion interactions were included *via* the Grimme DFT-D3 correction.^33^ Although AlGaSi_2_X_6_ is a free-standing monolayer with bonding dominated mainly by intralayer Al/Ga/Si–X interactions, D3 is not expected to qualitatively alter the main electronic or photocatalytic trends but its main role is to improve the optimized geometries and adsorption configurations by accounting for weak non-covalent contributions not fully captured by semilocal DFT.^34,35^ For Gibbs free-energy calculations, water-solvation effects were included using the implicit-solvation model VASPsol, which implements a continuum solvent description within plane-wave DFT and has been widely used to model solid–liquid interfacial energetics in VASP.^36^ A plane-wave kinetic-energy cutoff of 500 eV and a Monkhorst–Pack *k*-point mesh of 15 × 15 × 1 were used. Electronic self-consistency and ionic relaxation were converged to 10^−6^ eV in total energy and 0.01 eV Å^−1^ in residual forces, respectively. A vacuum region of 15 Å was introduced along the out-of-plane direction to suppress spurious interactions between periodic images of the monolayer. Lattice dynamical stability was assessed from phonon dispersions calculated with PHONOPY^37,38^ using a 3 × 3 × 1 supercell, and thermal robustness was further examined by *ab initio* molecular dynamics (AIMD)^39^ at 300 K for 5 ps. Further computational details and post-processing procedures are provided in the SI.

**Fig. 1:**
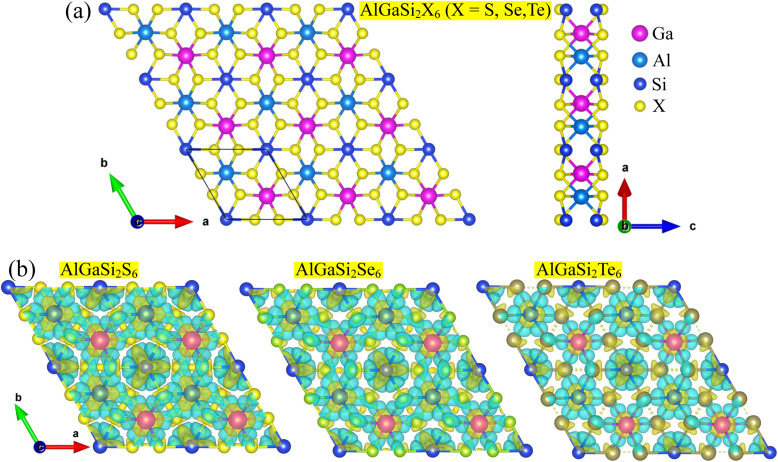
(a) Top and side views of the optimized AlGaSi_2_X_6_ (X = S, Se, Te) monolayer. Magenta, blue, cyan, and yellow spheres represent Ga, Al, Si, and X atoms, respectively. (b) Charge density difference isosurfaces for AlGaSi_2_S_6_, AlGaSi_2_Se_6_, and AlGaSi_2_Te_6_, where yellow and blue regions indicate charge accumulation and depletion, respectively.

Fig. 1(a) illustrates the optimized unit cell of AlGaSi_2_X_6_ (X = S, Se, Te) monolayers. In the top view, the atoms form a hexagonal unit cell, where Al (blue), Ga (magenta) and Si (cyan) occupy cation sites in a layered fashion, while the chalcogen atoms X (yellow) constitute the outermost planes. Each Si atom is tetrahedrally coordinated by four chalcogen atoms, whereas Al and Ga adopt trigonal coordination within the same X sublayer, giving rise to a compact three-cation, six-chalcogen framework. The side view highlights the multi-layered stacking along the *c* direction: X–Al–Si–Ga–X, yielding a puckered, five-atom-thick slab with mirror symmetry about the central Si–Si plane. During the structural optimization, a vacuum layer of 20 Å was used in the out-of-plane direction to ensure negligible interaction between periodic images.

## 3 Results and discussion

### 3.1 Structural properties

To examine the bonding characteristics, we analyzed the charge density difference of in AlGaSi_2_X_6_ (X = S, Se, Te), as shown in Fig. 1(b). For all three compositions, large charge accumulation (yellow) is observed along the Al–X, Ga–X and Si–X bonds, accompanied by charge depletion (blue) around the cation cores, which is consistent with a substantial charge transfer from electropositive Al/Ga/Si atoms toward the more electronegative chalcogen atoms. The charge redistribution becomes progressively more delocalized when going from S to Se to Te, reflecting the increasing spatial extent and polarizability of the heavier chalcogen p orbitals. This bonding picture confirms the mixed ionic–covalent character of the metal–chalcogen bonds and provides microscopic insight into how chemical substitution of X tunes the electronic structure and polarization of the AlGaSi_2_X_6_ monolayers.

**Fig. 2:**
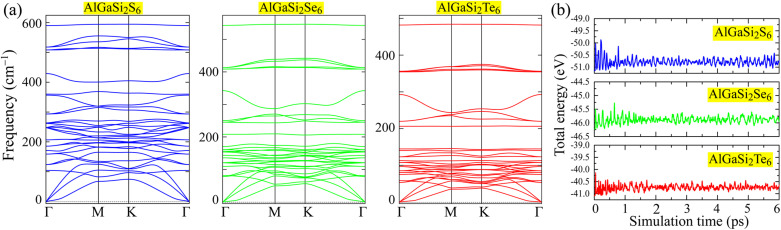
(a) Phonon dispersion curves of AlGaSi_2_S_6_, AlGaSi_2_Se_6_, and AlGaSi_2_Te_6_ monolayers along the high-symmetry path. (b) Time evolution of the total energy obtained from AIMD at 300 K for AlGaSi_2_S_6_, AlGaSi_2_Se_6_, and AlGaSi_2_Te_6_.

To assess dynamical stability, Fig. 2(a) shows the phonon dispersions of AlGaSi_2_S_6_, AlGaSi_2_Se_6_, and AlGaSi_2_Te_6_ along the high-symmetry path *Γ*–*M*–*K*–*Γ* of 2D hexagonal Brillouin zone, where *Γ* = (0, 0, 0), 
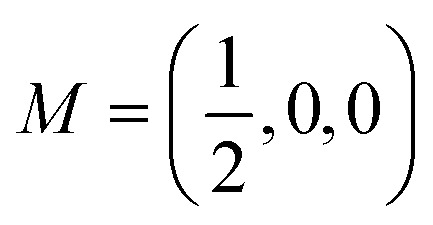
, and 
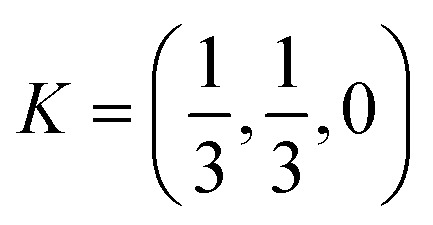
 in fractional reciprocal coordinates. For all three monolayers, no imaginary phonon frequencies are observed throughout the entire Brillouin zone, indicating the absence of lattice instabilities. Furthermore, the thermal stability of the AlGaSi_2_X_6_ monolayers was examined by AIMD at 300 K, and the resulting time evolution of the total energy is presented in Fig. 2(b). In each case, the total energy fluctuates around a nearly constant average value without any systematic drift during the entire MD simulation time of 5 ps. No bond breaking, layer reconstruction or structural collapse was observed in the MD trajectories. The combination of converging energy fluctuations and preserved crystal integrity confirms that all three AlGaSi_2_S_6_, AlGaSi_2_Se_6_, and AlGaSi_2_Te_6_ monolayers are thermally stable at room temperature.

Table 1 summarizes the key structural and electronic descriptors of the AlGaSi_2_X_6_ (X = S, Se, Te) monolayers, including the in-plane lattice constant *a*, interatomic distance *d*, layer thickness, PBE/HSE06 bandgaps, and cohesive energy *E*_coh_, respectively. Consistent with the increasing size of the chalcogen anion from S → Se → Te, the lattice constant increases monotonically (5.95, 6.29, and 6.85 Å), and the monolayer becomes slightly thicker (3.14, 3.33, and 3.58 Å). In line with this lattice expansion, the metal–chalcogen (Al–X and Ga–X) and Si–Si bond lengths show an overall elongation across the monolayers, indicating primary structural response to chalcogen substitution accommodated through the metal–chalcogen framework. Similarly, the electronic band gaps show a clear narrowing trend from S to Se to Te. At both the PBE and HSE06 levels, AlGaSi_2_S_6_ exhibits the largest gaps of *E*^PBE^_g_ = 1.85 eV and *E*^HSE06^_g_ = 2.79 eV, which decreases for AlGaSi_2_Se_6_ (1.49 and 2.32 eV) and reaches the smallest value for AlGaSi_2_Te_6_ (0.55 and 1.12 eV), as shown in Table 1. This behavior is in agreement with the stronger band-edge hybridization and narrower gap typically induced by heavier chalcogens.^40^

**Table 1:** Lattice constant *a*, interatomic distance *d*, thickness, PBE/HSE06 bandgap *E*_g_, and cohesive energy *E*_coh_ of AlGaSi_2_S_6_, AlGaSi_2_Se_6_, and AlGaSi_2_Te_6_

Structure	*a* (Å)	*d*_Si–X_ (Å)	*d*_Al–X_ (Å)	*d*_Ga–Y_ (Å)	*d*_Si−Si_ (Å)	Thickness (Å)	*E* ^PBE^_g_ (eV)	*E* ^HSE06^_g_ (eV)	*E*_coh_ (eV per atom)
AlGaSi_2_S_6_	5.95	2.14	2.47	2.51	2.22	3.14	1.85	2.79	−5.07
AlGaSi_2_Se_6_	6.29	2.30	2.61	2.65	2.26	3.33	1.49	2.32	−4.58
AlGaSi_2_Te_6_	6.85	2.52	2.82	2.87	2.30	3.58	0.55	1.12	−4.06

In addition, the cohesive energies *E*_coh_ are large in magnitude and negative for all three monolayers, indicating strong cohesion of the optimized 2D lattices. As shown in Table 1, AlGaSi_2_S_6_, AlGaSi_2_Se_6_, and AlGaSi_2_Te_6_ have *E*_coh_ of −5.07, −4.58, −4.06 eV per atom, respectively. This behavior implies a slight reduction in bonding strength with heavier chalcogens, while remaining sufficiently negative to support thermodynamic stability. Overall, the obtained lattice constants, bond lengths and cohesive energies of AlGaSi_2_X_6_ (X = S, Se, Te) are very similar to those reported for related III–IV–VI chalcogenide monolayers such as GaGeX_3_ and Ga_2_Ge_2_S_3_Se_3_, which also show lattice expansion and reduced cohesion when S is replaced by Se or Te.^41,42^ Likewise, the HSE06 band gaps from 2.79 to 1.12 eV follow the same chalcogen-mass trend and fall within the range (from 0.9 to 2.5 eV) reported for GaGeX_3_, Ga_2_Ge_2_X_3_Y_3_ and Ga_2_XY monolayers. This indicates that AlGaSi_2_X_6_ fits well into the broader family of group-III chalcogenides with suitable gaps for visible-light photocatalysis.^41–43^

The mechanical stability is also considered to further investigate the structural stability. Table 2 shows the elastic coefficients *C*_*ij*_, 2D Young's modulus *Y*_2D_, and Poisson's ratio *ν*_2D_ of AlGaSi_2_S_6_, AlGaSi_2_Se_6_, and AlGaSi_2_Te_6_, respectively. For a 2D hexagonal crystal, the Born–Huang stability criteria require that the in-plane stiffness tensor be positive definite, which can be written as *C*_11_ > 0 and *C*_66_ > 0 with *C*_66_ = (*C*_11_ − *C*_12_)/2 for hexagonal symmetry.^44,45^ All three monolayers satisfy these criteria, confirming mechanical stability against small deformations. The corresponding 2D Young's moduli of 93.65 Nm^−1^ for AlGaSi_2_S_6_, 78.62 Nm^−1^ for AlGaSi_2_Se_6_, and 61.16 Nm^−1^ for AlGaSi_2_Te_6_ indicate moderately stiff yet flexible monolayers, and show a clear softening trend from S to Te. Meanwhile, *ν*_2D_ remains within a narrow range (0.26–0.28), implying that chalcogen substitution primarily reduces in-plane rigidity rather than dramatically altering lateral contraction behavior.

**Table 2:** Elastic coefficients *C*_*ij*_, 2D Young's modulus *Y*_2D_, and Poisson's ratio *ν*_2D_ of AlGaSi_2_S_6_, AlGaSi_2_Se_6_ and AlGaSi_2_Te_6_

Structure	*C*_11_ (N m^−1^)	*C*_12_ (N m^−1^)	*C*_66_ (N m^−1^)	*Y*_2D_ (N m^−1^)	*ν*_2D_
AlGaSi_2_S_6_	100.59	26.43	37.08	93.65	0.26
AlGaSi_2_Se_6_	84.95	23.20	30.88	78.62	0.27
AlGaSi_2_Te_6_	66.51	18.85	23.83	61.16	0.28

### 3.2 Electronic characteristics

**Fig. 3:**
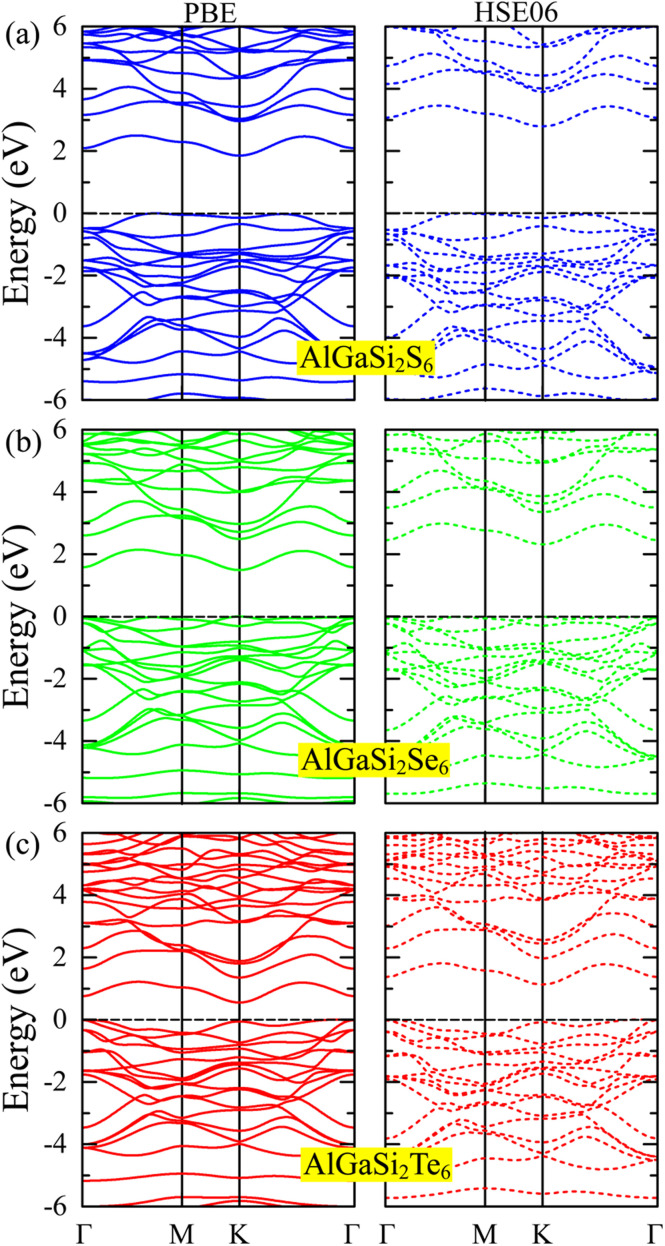
Electronic band structures of (a) AlGaSi_2_S_6_, (b) AlGaSi_2_Se_6_, (c) AlGaSi_2_Te_6_ calculated using PBE (left panels, solid lines) and HSE06 (right panels, dashed lines). The Fermi level is set to zero.

Fig. 3 shows the band structure of AlGaSi_2_X_6_ (X = S, Se, Te) monolayers. It is clear that all monolayers are semiconductors at both the PBE and HSE06 levels. In each case, the valence-band maximum (VBM) lies at an off-*Γ* point in the Brillouin zone, whereas the conduction-band minimum (CBM) sits very close to *Γ*. This misalignment of VBM and CBM shows that all three systems have an indirect band gap. Importantly, the indirect nature of the gap is preserved when going from PBE (solid lines) to HSE06 (dashed lines), where hybrid–functional corrections mainly open the gap without changing the band-edge *k*-points.

Comparing the three monolayers, the overall dispersion near the Fermi level is similar. The valence bands are relatively flat, especially near the VBM, indicating heavier holes, while the conduction bands around the CBM are more curved, implying lighter electrons and thus potentially higher electron mobility. As S is replaced by Se and then Te, both the PBE and HSE06 gaps shrink (from about 1.85/2.79 eV to 0.55/1.12 eV), consistent with the usual chalcogen trend.^40^ At the same time, the valence bands move upward and the conduction bands shift downward in energy, but their overall shapes and ordering remain almost unchanged, which suggests that chalcogen substitution mainly tunes the gap size rather than qualitatively altering the electronic structure.

**Fig. 4:**
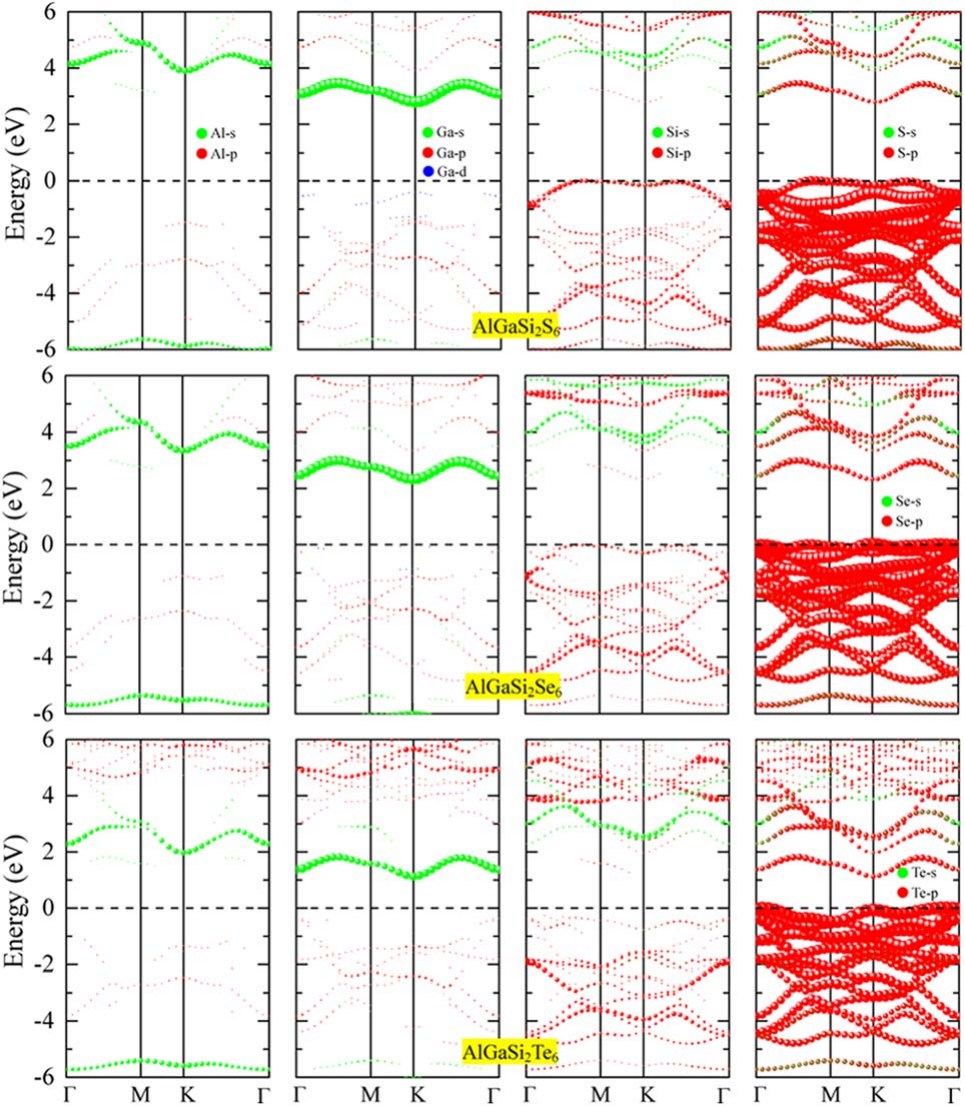
Atom-resolved band structures of AlGaSi_2_X_6_ monolayers with X = S (top row), Se (middle row) and Te (bottom row), projected onto (from left to right) Al, Ga, Si and chalcogen atoms. Green and red symbols denote s- and p-orbital contributions (with blue indicating Ga*-*d states). The Fermi level is set to zero.

The atom-projected band structures of AlGaSi_2_X_6_ (X = S, Se, Te) are shown in Fig. 4 to reveal orbital characters around the Fermi level. For all three monolayers, the valence-band region near the VBM is dominated by the chalcogen p states (S-p, Se-p, Te-p), with only minor admixture from Si-p and Ga-p orbitals. This confirms that the top of the valence band is mainly formed by X-p orbitals, consistent with the strong anion character of the bonding.

In contrast, the conduction-band edges are primarily composed of cation states: Al-s, Ga-s,p (with small Ga-d contributions), and Si-p. The CBM thus has mixed Al/Ga/Si character with relatively weak chalcogen contribution, which is favorable for electron transport along the cation framework. Comparing S, Se and Te, the chalcogen p bands systematically shift upward in energy and become slightly more dispersed, which explains the reduction of the band gap when going from AlGaSi_2_S_6_ to AlGaSi_2_Te_6_. Overall, the projected bands indicate predominantly anion-p-derived valence bands and cation-s/p-derived conduction bands, with heavier chalcogens bringing the valence edge closer to the Fermi level and narrowing the gap.

**Fig. 5:**
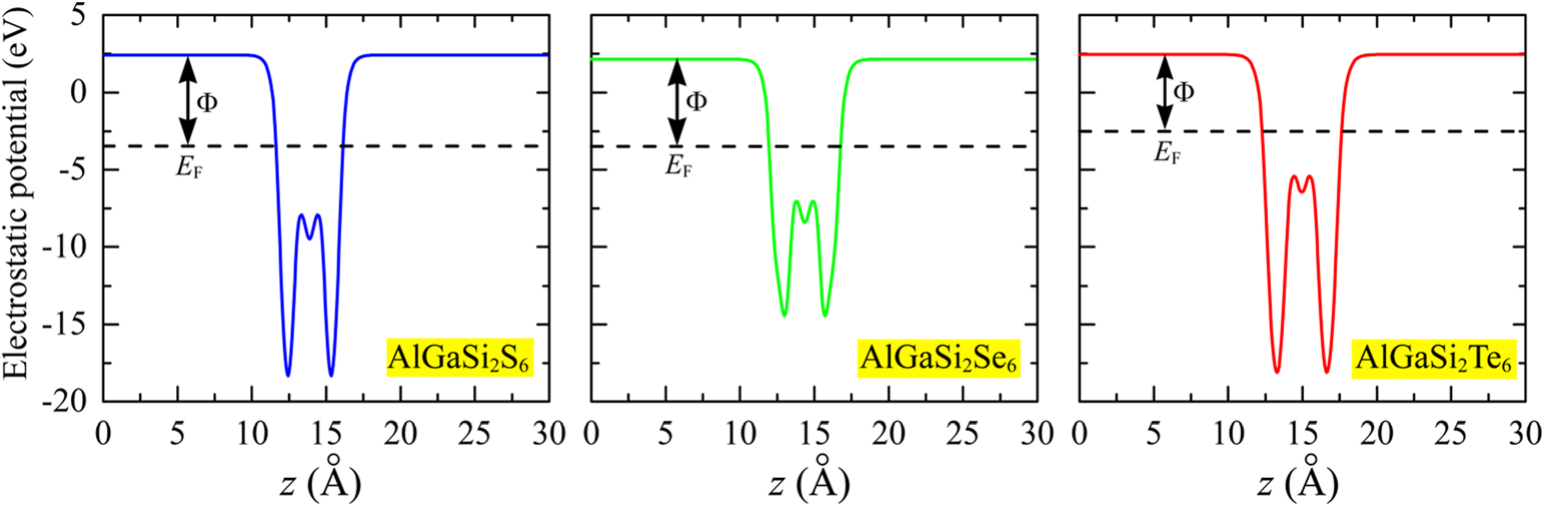
Planar-averaged electrostatic potential along the *z* direction for (left) AlGaSi_2_S_6_, (middle) AlGaSi_2_Se_6_, and (right) AlGaSi_2_Te_6_. The flat region at large |*z*| corresponds to the vacuum level, while the deep well marks the interior of the monolayer. The horizontal dashed line denotes the Fermi level *E*_F_, and the vertical arrow indicates the work function *Φ*.

Fig. 5 presents the planar-averaged electrostatic potential along the out-of-plane direction *z* for AlGaSi_2_S_6_, AlGaSi_2_Se_6_, and AlGaSi_2_Te_6_, respectively. In all three cases, the potential is nearly constant in the vacuum regions on both sides of the slab and exhibits a deep well in the interior of the monolayer, reflecting the strong confinement of electrons within the Al/Ga/Si–X framework. The very similar shapes of the potential profiles indicate that the overall electrostatic environment is preserved upon chalcogen substitution, while the small shift of *E*_F_ relative to the vacuum level reflects the modest tuning of the work function when going from S to Se to Te. This behavior reflects the systematic variation in chalcogen electronegativity and the resulting band-edge alignment, and it plays an important role in optimizing metal–contact properties and interfacial charge transfer in photocatalytic applications.

### 3.3 Photocatalytic performance

**Fig. 6:**
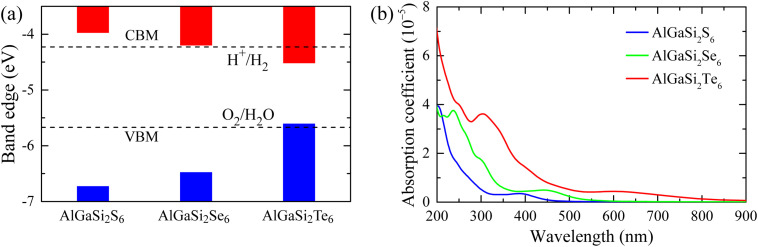
(a) Calculated band-edge positions of AlGaSi_2_X_6_ (X = S, Se, Te) relative to the vacuum level. Red (blue) bars denote the conduction-band minimum (valence-band maximum). The dashed horizontal lines mark the redox potentials of H^+^/H_2_ and O_2_/H_2_O at pH = 0. (b) Optical absorption coefficients of AlGaSi_2_X_6_ as a function of wavelength.

Fig. 6 compares the band-edge positions of AlGaSi_2_X_6_ (X = S, Se, Te) with the water redox potentials at pH = 0. For AlGaSi_2_S_6_ and AlGaSi_2_Se_6_ monolayers, the conduction-band minimum (CBM, red bars) lies above the H^+^/H_2_ and the valence-band maximum (VBM, blue bars) lies below the O_2_/H_2_O, so their band edges satisfy the thermodynamic requirement for overall water splitting. In addition, these S- and Se-based monolayers exhibit band gaps of 2.79 eV and 2.32 eV, respectively, which fall within the desirable 1.5–2.4 eV window for single-semiconductor photocatalysts and can thus provide sufficient photovoltage for overall water splitting.^46^ By contrast, in the AlGaSi_2_Se_6_ monolayer, the CBM is located below H^+^/H_2_ and the VBM lies above O_2_/H_2_O, and its much narrower band gap of 0.91 eV is too small to act as an efficient standalone light absorber for unbiased water splitting.

The corresponding absorption spectra in Fig. 6(b) show strong UV absorption for all three monolayers, with AlGaSi_2_Te_6_ exhibiting the largest and broadest response that extends furthest into the near-UV/visible region. This suggests that, while AlGaSi_2_Te_6_ is advantageous for harvesting a wider spectral range, AlGaSi_2_S_6_ and AlGaSi_2_Se_6_ are more promising as single-semiconductor photocatalysts for overall water splitting due to their more favorable band-gap values.

**Fig. 7:**
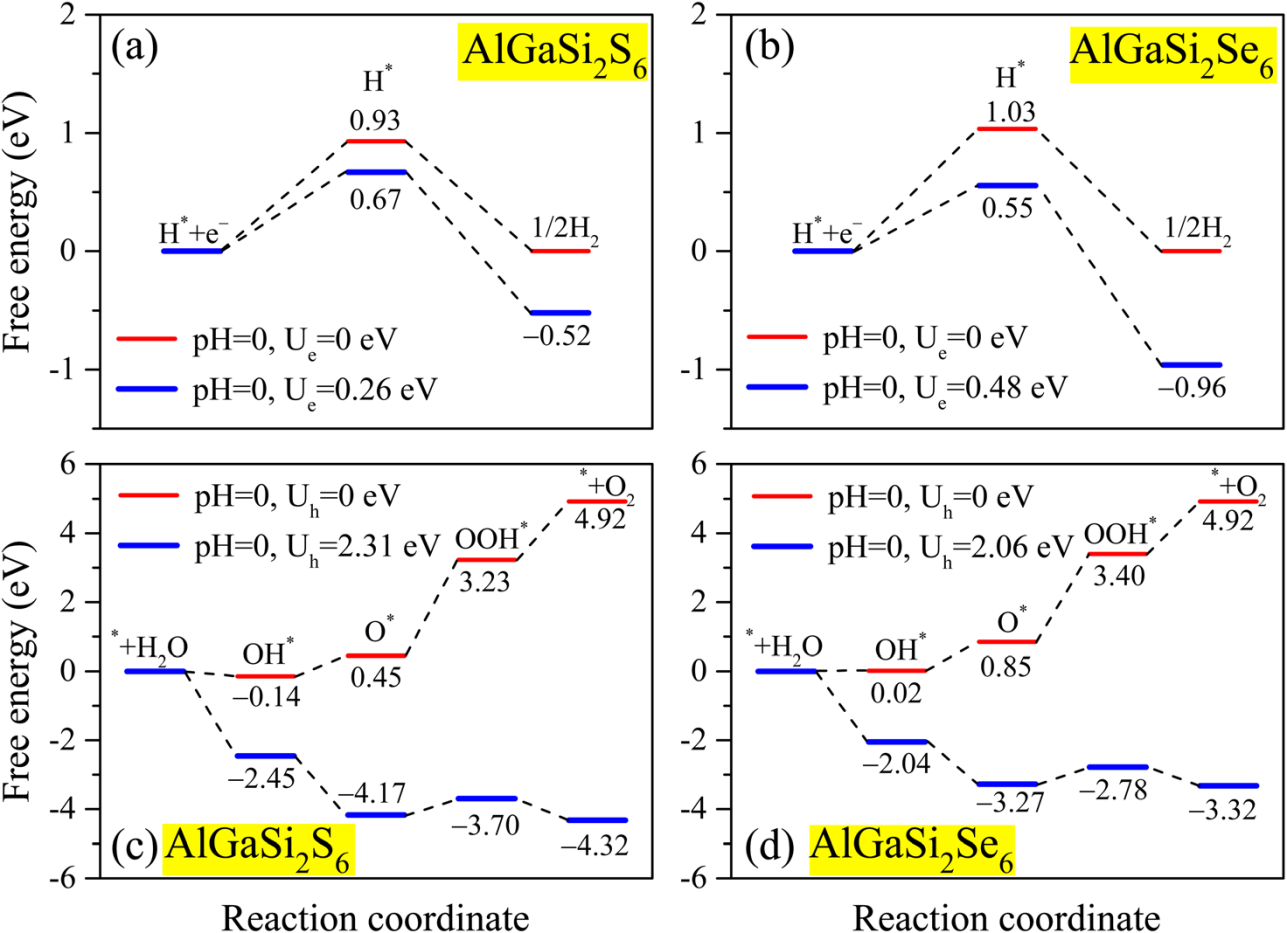
Gibbs free-energy diagrams for HER and OER on AlGaSi_2_X_6_ (X = S, Se) monolayers including implicit water solvation. (a) and (b) HER pathways on AlGaSi_2_S_6_ and AlGaSi_2_Se_6_, respectively. Red curves correspond to the equilibrium potential *U*_e_ = 0 V, while blue curves correspond to the electron quasi-Fermi levels at the CBM (*U*_e_ = 0.26 and 0.48 V, respectively). (c) and (d) OER pathways on AlGaSi_2_S_6_ and AlGaSi_2_Se_6_, respectively. Red curves are evaluated at *U*_h_ = 0 V, and blue curves at the hole quasi-Fermi levels equal to the VBM (*U*_h_ = 2.31 and 2.06 eV).

To identify the adsorption structure used in the Gibbs free-energy analysis, initial adsorbate configurations with different adsorption sites were examined for the H* intermediate. The adsorption energy was calculated as *E*_ads_ = *E*_AlGaSi_2_X_6_+H_ − *E*_AlGaSi_2_X_6__ − *E*_H_, where *E*_AlGaSi_2_X_6_+H_ is the total energy of the H-adsorbed AlGaSi_2_X_6_ system, *E*_AlGaSi_2_X_6__ is the total energy of the pristine AlGaSi_2_X_6_ monolayer, and *E*_H_ is the energy of an isolated H atom. The optimized geometries and corresponding adsorption energies of the candidate adsorption sites are provided in the SI (Table S1), and the most stable configuration, namely H adsorption at the Si site, was selected for the Gibbs free-energy calculations. Guided by the band-edge alignment analysis, we next investigated the HER thermodynamics of AlGaSi_2_S_6_ and AlGaSi_2_Se_6_ monolayers through their Gibbs free-energy profiles. Because HER and OER proceed at the solid–liquid interface, the aqueous environment can modify the Gibbs free energies of adsorbed intermediates and thus affect the overall free-energy profiles of the reaction pathways.^47,48^ Accordingly, to obtain a more realistic description of the HER/OER thermodynamics, we computed the Gibbs free-energy profiles in both vacuum and aqueous environments, as presented in Fig. 7(a) and (b), S1(a) and (b) in the SI. Overall, water solvation induces a systematic downward shift in the absolute Gibbs free energies of the adsorbed intermediates, small for H* in HER (by about 0.16–0.18 eV) but considerably greater for the oxygenated OER species (typically by 0.6–0.8 eV), while preserving the same overall qualitative reaction trends on both monolayers. At the equilibrium potential *U*_e_ = 0 V (red curves), the adsorption step H^+^ + e^−^ → H* is clearly uphill, with Δ*G*_H*_ = 0.93 eV for AlGaSi_2_S_6_ and 1.03 eV for AlGaSi_2_Se_6_. This indicates relatively weak H binding and low intrinsic HER activity under *U*_e_ = 0 conditions. Within the photoexcited scenario (blue curves), where the electron chemical potential is shifted to the CBM (*U*_e_ = 0.26 eV for AlGaSi_2_S_6_ and 0.48 eV for AlGaSi_2_Se_6_), the Gibbs free energy is reduced to 0.67 and 0.55 eV, respectively. However, H* formation remains the potential-determining step. Thus, both monolayers can in principle catalyze HER under photoexcitation, with AlGaSi_2_Se_6_ requiring a slightly smaller driving force due to its lower maximum Δ*G*. This is consistent with the general expectation that an efficient HER catalyst should exhibit Δ*G*_H*_ close to zero.^49^

For the OER, the free-energy pathways along the conventional four proton–coupled electron-transfer pathway H_2_O* → OH* → OOH* → O_2_ (ref. 50) were summarized in Fig. 7(c) and (d). The optimized adsorption geometries of the OH*, O*, and OOH* intermediates employed in the OER free-energy calculations are provided in the SI to improve transparency and reproducibility, consistent with recent DFT reporting practice for 2D OER/ORR catalysts.^51–53^ In all cases considered here, the OOH* intermediate converged to a stable adsorbed configuration during structural optimization. At *U*_h_ = 0 V (red curves), multiple steps are endergonic, and the OOH* formation emerges as the largest uphill barrier, which is a well-known consequence of the strong scaling relations among the three oxygen-containing intermediates (OH*, O*, OOH*) that impose an intrinsic energetic penalty on OER catalysts.^54,55^ When *U*_h_ is shifted to the VBM (2.31 eV for AlGaSi_2_S_6_ and 2.06 eV for AlGaSi_2_Se_6_), as shown in blue, all elementary steps become downhill, indicating that photogenerated holes at the VBM can supply sufficient thermodynamic driving force for OER on these surfaces. The corresponding overpotentials are approximately *η*_OER_ ≈ *U*_h_ − 1.23 V,^54^ giving about 1.08 V for AlGaSi_2_S_6_ and 0.83 V for AlGaSi_2_Se_6_, which suggests that the Se-based monolayer is somewhat more favorable for OER, although both still require substantial overpotentials compared with the thermodynamic limit. Overall, these free-energy landscapes indicate that AlGaSi_2_S_6_ and AlGaSi_2_Se_6_ can in principle drive both HER and OER under illumination, with HER being easier than OER and AlGaSi_2_Se_6_ offering slightly more balanced reaction energetics.

To further benchmark the practical photocatalytic potential, we estimated the theoretical solar-to-hydrogen (STH) efficiency for AlGaSi_2_S_6_ and AlGaSi_2_Se_6_ using the commonly adopted AM1.5G spectral-integration descriptor. Table 3 reports the HER and OER overpotentials *χ*(H_2_) and *χ*(O_2_), light absorption efficiency *η*_abs_, charge-carrier utilization *η*_cu_ and solar-to-hydrogen (STH) efficiency *η*_STH_ of AlGaSi_2_S_6_ and AlGaSi_2_Se_6_ monolayers, respectively. In comparison to AlGaSi_2_S_6_, the Se-containing monolayer exhibits smaller *χ*(H_2_) and *χ*(O_2_), which increases *η*_cu_ because a larger fraction of absorbed photon energy can be converted into the chemical free energy of water splitting. At the same time, AlGaSi_2_Se_6_ shows a markedly higher *η*_abs_, consistent with stronger solar harvesting. As a result, *η*_STH_ is achieved 3.90% for AlGaSi_2_S_6_ and 10.68% for AlGaSi_2_Se_6_, placing AlGaSi_2_Se_6_ in the performance range often regarded as promising in theoretical screenings of 2D water-splitting photocatalysts.^56–58^

**Table 3:** HER and OER overpotentials *χ*(H_2_) and *χ*(O_2_), light absorption efficiency *η*_abs_, charge-carrier utilization *η*_cu_ and solar-to-hydrogen (STH) efficiency *η*_STH_ of AlGaSi_2_S_6_ and AlGaSi_2_Se_6_ monolayers

Structure	*χ*(H_2_)	*χ*(O_2_)	*η*_abs_ (%)	*η*_cu_ (%)	*η*_STH_ (%)
AlGaSi_2_S_6_	0.48	1.08	9.95	39.23	3.90
AlGaSi_2_Se_6_	0.26	0.83	23.91	44.68	10.68

### 3.4 Intrinsic charge-carrier transport

**Fig. 8:**
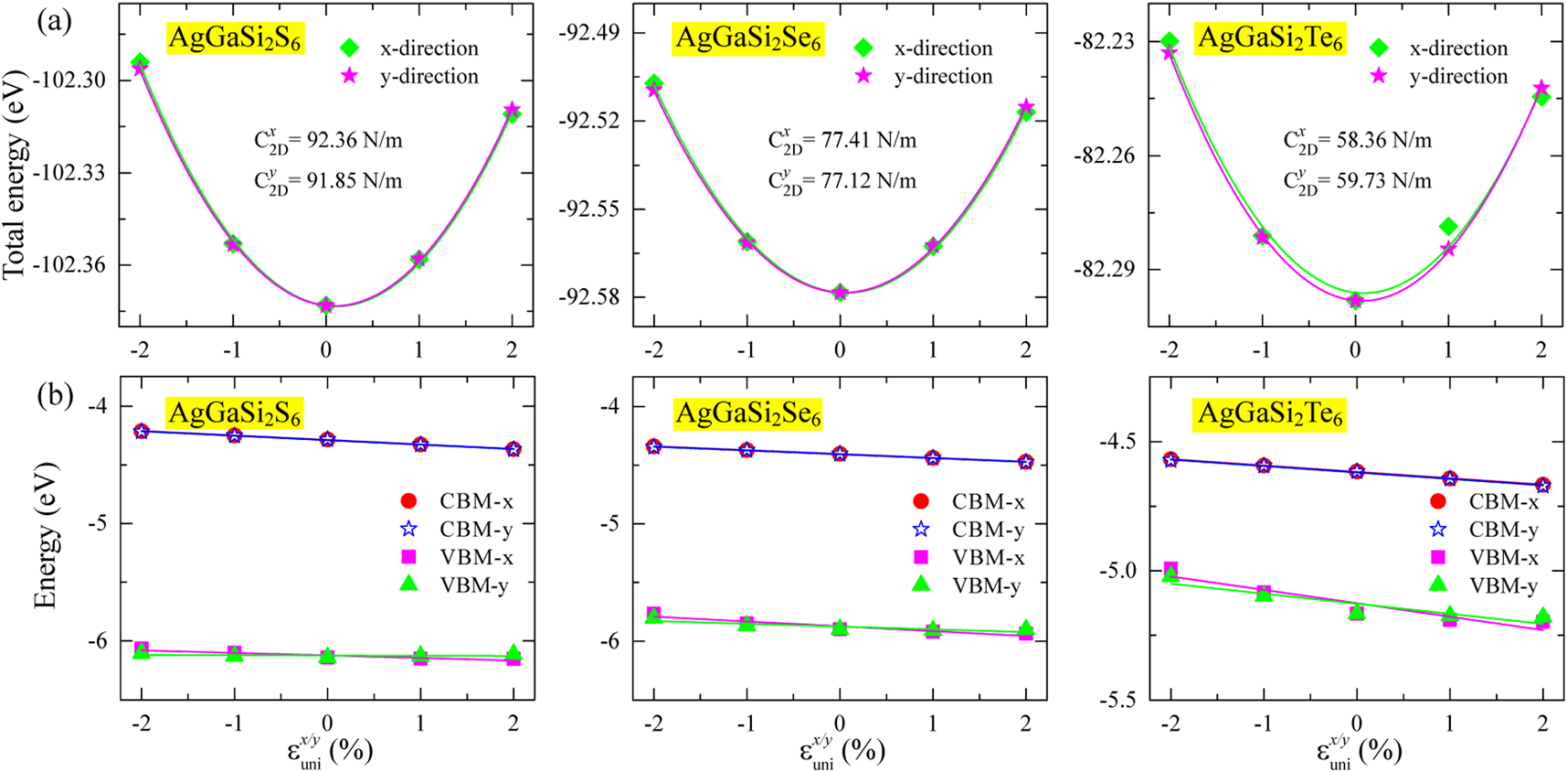
(a) Total energy of AlGaSi_2_X_6_ (X = S, Se, Te) as a function of uniaxial strain *ε*_uni_^*x*/*y*^ along the *x* and *y* directions. In-plane elastic constants *C*_2D_^*x*/*y*^ are obtained from quadratic fits. (b) Strain dependence of the conduction-band minimum (CBM) and valence-band maximum (VBM) along *x* and *y*.

To complement the band alignment and reaction thermodynamics analysis, we further examine intrinsic carrier transport, since carrier mobility and diffusion critically impact recombination losses and the availability of electrons and holes at catalytic sites. Fig. 8(a) shows the variation of the total energy of AlGaSi_2_X_6_ (X = S, Se, Te) under small uniaxial strains *ε*_uni_^*x*/*y*^ applied along the in-plane *x* and *y* directions. For each case, the energy–strain relation is well captured by a quadratic form within ±2%, consistent with the expected harmonic regime used to extract 2D in-plane elastic constants *C*_2D_^*x*/*y*^ from DFT. The results show that AlGaSi_2_S_6_ is the stiffest (*C*_2D_ ≈ 92 Nm^−1^), AlGaSi_2_Se_6_ is softer (*C*_2D_ ≈ 77 Nm^−1^), AlGaSi_2_Te_6_ is the softest with the smallest *C*_2D_ ≈ 58–60 Nm^−1^ and a noticeable anisotropy between *x* and *y* directions.

Fig. 8(b) presents the strain dependence of the band edges (CBM and VBM). Across all three monolayers, the band edges shift smoothly and approximately linearly with *ε*_uni_^*x*/*y*^, which is the defining behavior assumed in deformation-potential (DP) theory.^59^ The nearly linear and only weakly anisotropic behavior over ±2% imply that moderate mechanical deformation provides a controllable means to tune band edges without inducing abrupt electronic transitions within the explored range. Such strain-tolerant, continuously tunable band-edge behavior is widely viewed as advantageous for flexible electronics and strain-engineered 2D devices.

The key transport quantities extracted within the 2D DP framework, including effective masses m*, in-plane elastic constants *C*_2D_, deformation potentials *E*_d_ and carrier mobilities *µ*_2D_ along the *x* and *y* directions, are summarized in Table 4. It should be noted that the listed effective masses were obtained from parabolic fitting of the frontier bands near the relevant band extrema (CBM and VBM) used in the deformation-potential analysis, rather than being evaluated separately at all high-symmetry points of the Brillouin zone. Overall, the electron effective masses are light and essentially isotropic (0.30 *m*_0_) together with nearly identical *C*_2D_ and *E*_d_, which yields high and almost direction-independent electron mobility nearly to 1.7 × 10^3^ cm^2^ V^−1^ s^−1^. In contrast, hole transport is more anisotropic. Even with comparable hole effective masses (0.45–0.47 *m*_0_), the much larger |*E*_d_| along *x* suppresses the mobility, giving a threefold lower *µ*_2D_ than in the *y* direction (473 *vs.* 1382 cm^2^ V^−1^ s^−1^). Overall, AlGaSi_2_Se_6_ is predicted to exhibit high intrinsic carrier transport, with particularly favorable mobility along the high-mobility in-plane direction, suggesting an encouraging feature for suppressing recombination and facilitating carrier extraction in photocatalytic operation.

**Table 4:** Effective masses m* (*m*_0_), elastic moduli *C*_2D_, deformation potentials *E*_d_, and carrier mobilities *m*_2D_ along the *x*- and *y*-directions for the AlGaSi_2_Se_6_ monolayer

Carrier type		m* (*m*_0_)	*C*_2D_ (N m^−1^)	*E*_d_ (eV)	*µ*_2D_ (cm^2^ V^−1^ s^−1^)
Electron	*x*	0.30	77.41	−3.28	1683.89
	*y*	0.30	77.12	−3.26	1698.31
Hole	*x*	−0.45	77.41	−4.12	472.68
	*y*	−0.47	77.12	−2.36	1382.02

## 4 Conclusion

We systematically investigated the quaternary 2D chalcogenide monolayers AlGaSi_2_X_6_ (X = S, Se, Te) using first-principles calculations, focusing on structural stability, electronic structure, carrier transport, and photocatalytic properties. All three compositions are predicted to be robust 2D lattices, supported by dynamical and thermal stability as well as strongly negative cohesive energies. Chalcogen substitution from S/Se to Te expands the lattice and softens the sheet while driving a large band gap narrowing. Overall, AlGaSi_2_S_6_ and AlGaSi_2_Se_6_ remain the most promising for single-absorber water splitting because they can provide the required thermodynamic driving force in the commonly discussed band-gap window for overall water splitting. Consistent with this, the calculated HER/OER free-energy landscapes support photoassisted operation for the S- and Se-based monolayers, and the spectral-integration metric yields STH efficiencies of 3.90% for AlGaSi_2_S_6_ and 10.68% for AlGaSi_2_Se_6_, highlighting AlGaSi_2_Se_6_ as the most attractive candidate for overall water-splitting photocatalysis.

## Conflicts of interest

There are no conflicts of interest to declare.

## Supplementary Material

RA-016-D6RA00973E-s001

## Data Availability

The data supporting the findings of this study are available from the corresponding authors upon reasonable request. Supplementary information (SI) is available. See DOI: https://doi.org/10.1039/d6ra00973e.
