# Supplementary material for: First-principles study of AlGaSi2X6 (X = S, Se, Te) monolayers: structural, electronic, transport and photocatalytic properties
Source: RSC Adv. 2026 May 6;16(25):23120–8. doi: 10.1039/d6ra00973e (PMC13147219; doi:10.1039/d6ra00973e)
Supplement: RA-016-D6RA00973E-s001 [file RA-016-D6RA00973E-s001.pdf]

Supplementary Information (SI) for RSC Advances.
This journal is © The Royal Society of Chemistry 2026

Supporting Information

First-principles Study of $AlGaSiS_2X_6$ (X = S, Se, Te) Monolayers: Structural, Electronic, Transport and Photocatalytic Properties

Thi H. Ho[1,2,*], Tuan V. Vu[1,2*], A. I. Kartamyshev[1,2], Dat D. Vo[1,2], Duy Khanh Nguyen[1,2] and Nguyen N. Hieu[3,4]

[1]Laboratory for Computational Physics, Institute for Computational Science and Artificial Intelligence, Van Lang University, Ho Chi Minh City, Vietnam

[2]Faculty of Mechanical, Electrical, and Computer Engineering, Van Lang School of Technology, Van Lang University, Vietnam

[3]Institute of Research and Development, Duy Tan University, Da Nang 550000, Vietnam

[4]Faculty of Natural Sciences, Duy Tan University, Da Nang 550000, Vietnam

[*]Corresponding authors

Emails: thi.hohuynh@vlu.edu.vn (T. H. Ho), tuan.vu@vlu.edu.vn (Tuan V. Vu)

Detailed Computational Methods

To further assess the thermodynamic stability of $AlGaSi_2X_6$ monolayers, we considered the cohesive energy, defined as the energy difference between its total energy and the combined energies of its individual components, as follows:

$$E_{coh} = \frac{E_{Al} + E_{Ga} + 2E_{Si} + 6E_X - E_{tot}}{10}$$

where E_{tot} is the total energy of the relaxed system. E_{Al}, E_{Ga}, E_{Si}, and E_X are the total energy of isolated Ga, Si, S, Se or Te atom, respectively. $E_{coh} < 0$ indicates favorable bonding strength.

A key elementary step in photocatalytic water splitting is the hydrogen evolution reaction (HER), which is governed by the adsorption of reaction intermediates H^* on catalytically active sites. To quantify the HER activity, we evaluated the Gibbs free energy of hydrogen adsorption ΔG_H on the $AlGaSi_2X_6$ monolayers, as follows[1-3]:

$$\Delta G_H = \Delta E_H + 0.24\ eV$$

ΔE_H is defined as:

$$\Delta E_H = E_{AlGaSiX_6 + H} - E_{AlGaSiX_6} - 0.5E_{H_2}$$

where $E_{AlGaSiX_6 + H}$, $E_{AlGaSiX_6}$, and E_{H_2} denote the total energies of the H-adsorbed system, pristine monolayer, and isolated H_2 molecule, respectively.

For the oxygen evolution reaction (OER), we evaluated the Gibbs free-energy change for each elementary proton–coupled electron-transfer step along the conventional four-step mechanism,

(1) $H_2O_{(l)} + {}^* \rightarrow OH^* + (H^+ + e^-)$
(2) $OH^* \rightarrow O^* + (H^+ + e^-)$
(3) $H_2O_{(l)} + O^* \rightarrow OOH^* + (H^+ + e^-)$
(4) $OOH^* \rightarrow {}^* + O_{2(g)} + (H^+ + e^-)$

Here, * denotes an empty surface adsorption site, and OH^*, O^*, and OOH^* are adsorbed intermediates. All adsorption geometries were optimized using one adsorbate per surface supercell.

Within the computational hydrogen electrode (CHE) framework, the free-energy change of step i at an electrode potential U is computed as[4],

$$\Delta G_i = \Delta E_i + \Delta E_{ZPE} - T\Delta S_i - eU$$

where ΔE_i is is the DFT reaction energy, ΔE_{ZPE} and ΔS are the corresponding changes in zero-point energy and entropy, respectively. The term $-eU$ accounts for the work associated with transferring one electron at potential U. In the CHE model, the chemical potential of the $(H^+ + e^-)$ pair is referenced to $\frac{1}{2}H_2$ at $U = 0$ and $pH = 0$. Th pH correction can be added when required.

To quantify in-plane transport, we estimated the intrinsic carrier mobility using the 2D Bardeen–Shockley deformation-potential (DP) formalism[5]. Small uniaxial strains $\varepsilon_{uni}^{x/y}$ were applied along the x or y direction, and at each strain we recalculated the total energy E_{total} and the relevant band-edge energy E_{edge}. The strain–energy relation $E_{tot}(\varepsilon)$ was sampled from -2% to 2% and fitted with a quadratic function, from which the in-plane 2D elastic modulus was obtained as

$$C_{2D}^{x/y} = \frac{1}{A_0} \frac{\partial^2 E_{total}}{\partial\left(\varepsilon_{uni}^{x/y}\right)^2}$$

where A_0 s the equilibrium cell area. Within the DP framework, the mobility along a given direction is then evaluated by,

$$\mu_{2D} = \frac{e\hbar^3 C_{2D}}{k_B T m^* m_d^* E_d^2}$$

with e the elementary charge, $\hbar$ the reduced Planck constant, k_B the Boltzmann constant, and T the temperature. Here, $m_{x/y}^*$ is the transport effective mass along x/y, $m_d^* = \sqrt{m_x^* m_y^*}$ is the density-of-states effective mass Effective masses, and $E_d = \partial E_{edge}/\partial \varepsilon$ is the deformation-potential constant extracted from the linear slope of the band edge with strain. The effective masses were obtained from parabolic fitting of the band dispersion near the extrema,

$$m^* = \left(\frac{\hbar^2\partial^2 E}{\partial k^2}\right)^{-1}$$

The theoretical solar-to-hydrogen (STH) conversion efficiency provides a convenient upper-bound metric for how effectively a semiconductor photocatalyst could transform incident sunlight into the chemical free energy stored in H_2. Following the widely used AM1.5G spectral-integration descriptor, we express the overall efficiency as the product of the light-absorption efficiency η_{abs} and the carrier-utilization efficiency η_{cu}[6]:

$$\eta_{STH} = \eta_{abs} \times \eta_{cu}$$

The absorption term η_{abs} measures the fraction of the solar power carried by photons energetic enough to be absorbed across the band gap,

$$\eta_{abs} = \frac{\int_{E_g}^{\infty} P(\hbar\omega)\, d(\hbar\omega)}{\int_{0}^{\infty} P(\hbar\omega)\, d(\hbar\omega)}$$

where $P(h\omega)$ denotes the AM1.5G solar spectral irradiance as a function of photon energy $\hbar\omega$ and band gap E_g of photocatalyst.

The carrier-utilization efficiency accounts for the portion of the absorbed photon energy that can be converted into the Gibbs free energy required for overall water splitting:

$$\eta_{cu} = \frac{\Delta G_{H_2O} \int_{E}^{\infty} \frac{P(\hbar\omega)}{\hbar\omega} d(\hbar\omega)}{\int_{E_g}^{\infty} P(\hbar\omega) d(\hbar\omega)}$$

where ΔG_{H_2O} = 1.23 eV is the minimum free-energy cost per transferred electron under standard conditions. The lower photon-energy threshold E incorporates additional energetic losses associated with the kinetic overpotentials for HER $\chi(H_2)$ and OER $\chi(O_2)$, through:

$$E = \begin{cases} E_g & \chi(H_2) \geq 0.2 \ and\ \chi(O_2) \geq 0.6 \\ E_g + 0.2 - \chi(H_2) & \chi(H_2) < 0.2 \ and\ \chi(O_2) \geq 0.6 \\ E_g + 0.6 - \chi(O_2) & \chi(H_2) \geq 0.2 \ and\ \chi(O_2) < 0.6 \\ E_g + 0.8 - \chi(H_2) - \chi(O_2) & \chi(H_2) < 0.2 \ and\ \chi(O_2) < 0.6 \end{cases}$$

Here, the commonly adopted reference values of 0.2 eV (HER) and 0.6 eV (OER) represent typical kinetic penalties used in theoretical screening of overall water-splitting photocatalysts.

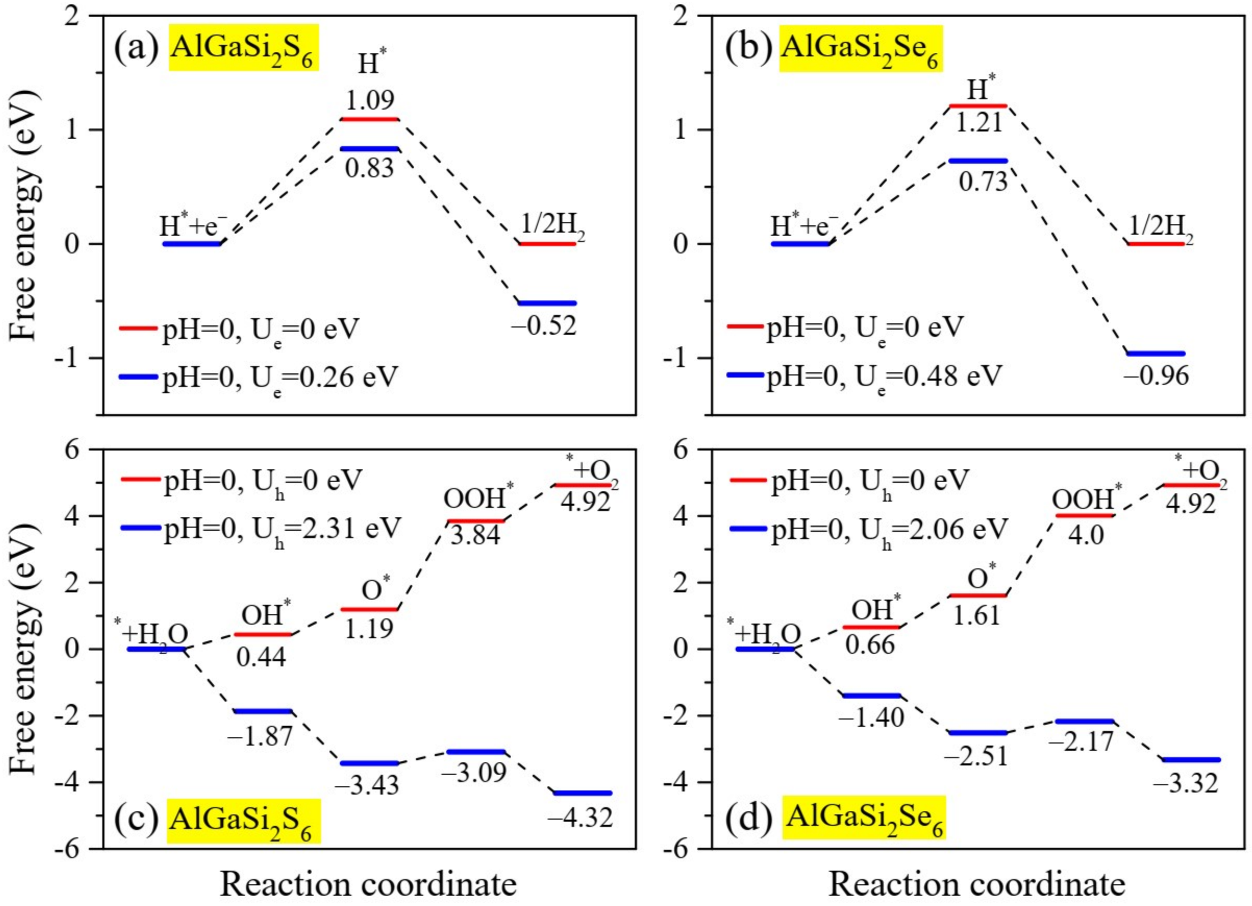

Figure S1. Gibbs free-energy diagrams for HER and OER on $AlGaSi_2X_6$ (X = S, Se) monolayers under vacuum condition. (a)-(b) HER pathways on $AlGaSi_2S_6$ and $AlGaSi_2Se_6$, respectively. Red curves correspond to the equilibrium potential Ue = 0 V, while blue curves correspond to the electron quasi-Fermi levels at the CBM (Ue = 0.26 and 0.48 V, respectively). (c)-(d) OER pathways on $AlGaSi_2S_6$ and $AlGaSi_2Se_6$, respectively. Red curves are evaluated at U_h = 0 V, and blue curves at the hole quasi-Fermi levels equal to the VBM (U_h = 2.31 and 2.06 eV).

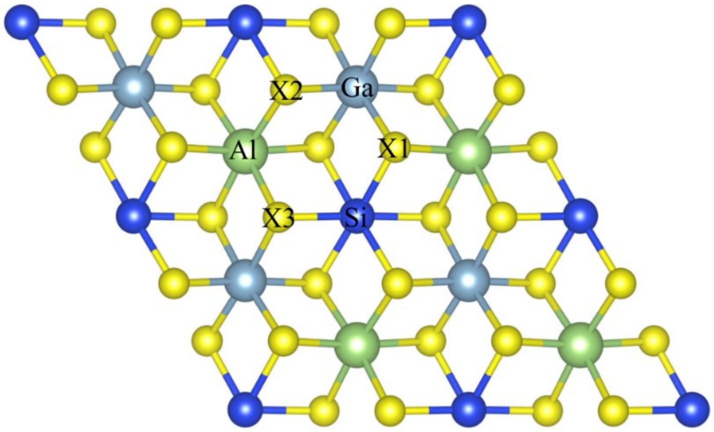

Figure S2. Candidate adsorption sites considered for the H^* intermediate on the $AlGaSi_2X_6$ monolayer. Al, Ga, Si, X1, X2, and X3 denote the initial adsorption positions used in the adsorption-site screening.

Table S1. Corresponding adsorption energies E_{ads} for the H^* intermediate at different adsoprtion sites.

**Site**	E_{ads} **(eV)**	
	**$AlGaSi_2S_6$**	**$AlGaSi_2Se_6$**
Al	2.06	1.79
Ga	2.23	1.28
**Si**	**0.69**	**0.80**
X1	1.02	1.03
X2	1.08	0.90
X3	1.06	0.90

Optimized pristine $AlGaSi_2S_6$, $AlGaSi_2Se_6$, and $AlGaSi_2Te_6$ structures in CONTCAR format.

```
AlGaSi2S6
   1.00000000000000
     5.9474768449813338    0.0000000000000000   -0.0000000000000000
    -2.9737384224906678    5.1506660361894347    0.0000000000000000
     0.0000000000000017    0.0000000000000030   27.7635467867882433
  Si              Ga              S               Al
     2     1     6     1
Direct
  0.0000000000000000  0.0000000000000000  0.5400481375662665
  0.0000000000000000  0.0000000000000000  0.4599519084337374
  0.6666666870000029  0.3333333429999996  0.5000000120000010
  0.0065193257314825  0.3550116052130352  0.4434007304485856
  0.0065193257314824  0.6515077205184474  0.5565992695514145
  0.6449883947869651  0.6515077205184474  0.4434007304485856
  0.3484922794815526  0.3550116052130352  0.5565992695514145
  0.3484922794815526  0.9934806742685175  0.4434007304485856
  0.6449883947869651  0.9934806742685175  0.5565992695514145
  0.3333333429999996  0.6666666870000029  0.5000000120000010
```

```
AlGaSi2Se6
   1.00000000000000
     6.2943128778221418   -0.0000000000000000    0.0000000000000000
```

-3.1471564389110722 5.4510348515802045 0.0000000000000000

0.0000000000000018 0.0000000000000032 28.7129522803659576

Si Ga Se Al

2 1 6 1

Direct

0.0000000000000000 0.0000000000000000 0.5392595391280547

0.0000000000000000 0.0000000000000000 0.4607405068719492

0.6666666870000029 0.3333333429999996 0.5000000120000010

0.0059321989523177 0.3572970170739419 0.4419700495999449

0.0059321989523179 0.6486351818783758 0.5580299504000552

0.6427029829260579 0.6486351818783758 0.4419700495999449

0.3513648181216242 0.3572970170739419 0.5580299504000552

0.3513648181216245 0.9940678010476822 0.4419700495999449

0.6427029829260578 0.9940678010476822 0.5580299504000552

0.3333333429999996 0.6666666870000029 0.5000000120000010

AlGaSi2Te6

1.00000000000000

6.8476855424346663 -0.0000000000000002 -0.0000000000000000

-3.4238427712173340 5.9302696368991077 0.0000000000000001

0.0000000000000020 0.0000000000000036 29.9126920714541811

Si Ga Te Al

2 1 6 1

Direct

0.0000000000000000 0.0000000000000000 0.5384029979754724

0.0000000000000000 0.0000000000000000 0.4615970480245313

0.6666666870000029 0.3333333429999996 0.5000000120000010

0.0049809587403582 0.3584277956993634 0.4401187153853054

0.0049809587403570 0.6465531630409941 0.5598812846146943

0.6415722043006371 0.6465531630409941 0.4401187153853054

0.3534468369590053 0.3584277956993634 0.5598812846146943

0.3534468369590048 0.9950190412596424 0.4401187153853054

0.6415722043006377 0.9950190412596424 0.5598812846146943

0.3333333429999996 0.6666666870000029 0.5000000120000010

Optimized structures of the OH^*, O^*, and OOH^* intermediates on the $AlGaSi_2X_6$ in CONTCAR format.

AlGaSi2S6-O

1.00000000000000

11.8949537277000008   0.0000000000000000   0.0000000000000000

-5.9474768639000004   10.3013321050000002   0.0000000000000000

0.0000000000000000   0.0000000000000000   27.7635459900000008

Si Ga S Al O

8 4 24 4 1

Direct

0.9967675531516272 0.9964627986400614 0.5377919698806727

0.0035372013599390	0.5003047545115663	0.5377919698806727
0.4996952454884341	0.0032324468483728	0.5377919698806727
0.5000000000000000	0.5000000000000000	0.5894526393688850
0.9949955251887491	0.9960378872648921	0.4574135743720895
0.0039621127351081	0.4989576379238571	0.4574135743720895
0.5010423620761432	0.0050044748112508	0.4574135743720895
0.5000000000000000	0.5000000000000000	0.4871048025109310
0.3232919326898500	0.1622227931566628	0.4940799623827686
0.3389308774668176	0.6767080823101548	0.4940799623827686
0.8333333440000033	0.1666666710000015	0.4990356965037819
0.8377772218433417	0.6610691375331866	0.4940799623827686
0.9998390014555080	0.1743095432770388	0.4410210632995520
0.0076566327426239	0.6762690815286582	0.4398060064714338
0.5041051785568503	0.1824102993727189	0.4413954401150811
0.5018785405495633	0.6654584240577202	0.4470454904615989
0.0065965119015800	0.3254733280175220	0.5539487391123996
0.9994324873791522	0.8209343982571629	0.5528883615362880
0.5020477330054886	0.3454754457385743	0.5444985227363561
0.5016803718774393	0.8283707835086273	0.5543783249654198
0.3345415759422792	0.3364201164918421	0.4470454904615989
0.3237309184713413	0.8313875512139649	0.4398060064714338
0.8256904567229618	0.3255294581784688	0.4410210632995520
0.8175897006272809	0.8216948791841306	0.4413954401150811

0.1716292164913726 0.1733095883688116 0.5543783249654198
0.1790656017428376 0.6784980891219902 0.5528883615362880
0.6745266719824784 0.1811231838840582 0.5539487391123996
0.6545245542614256 0.6565722872669137 0.5444985227363561
0.1783051208158689 0.4958948214431497 0.4413954401150811
0.1686124487860349 0.9923433672573755 0.4398060064714338
0.6635798835081576 0.4981214594504372 0.4470454904615989
0.6744705418215308 0.0001609985444924 0.4410210632995520
0.3434277127330858 0.4979522669945117 0.5444985227363561
0.3215019108780104 0.0005675126208478 0.5528883615362880
0.8266904116311881 0.4983196281225610 0.5543783249654198
0.8188768161159422 0.9934034880984204 0.5539487391123996
0.1651272399773994 0.3254897053224492 0.4964015965607221
0.1666666710000015 0.8333333440000033 0.4977621552894979
0.6745103096775560 0.3396375516549547 0.4964015965607221
0.6603624633450493 0.8348727750226052 0.4964015965607221
0.5000000000000000 0.5000000000000000 0.6446378306437818

AlGaSi2S6-OH

1.00000000000000

11.8949537277000008 0.0000000000000000 0.0000000000000000
-5.9474768639000004 10.3013321050000002 0.0000000000000000
0.0000000000000000 0.0000000000000000 27.7635459900000008

Si	Ga	S	Al	O	H
8	4	24	4	1	1

Direct

0.9985991979825907	0.9985478679259507	0.5374877984377294
0.9997981560455329	0.4994209468516493	0.5374053171827943
0.4991854727360594	0.9997290172139882	0.5373895929169973
0.5010850352429607	0.5011124002439108	0.5662803624882413
0.9981669387841344	0.9983411188259517	0.4571318835082967
0.0000817921440839	0.4992271337599207	0.4570720249710061
0.4994177636010135	0.0001868977154479	0.4570470696038377
0.4990489177103390	0.4991028231144504	0.4725756360313444
0.3300734765213941	0.1643095688019608	0.4967015547890092
0.3333672850197004	0.6689592816567805	0.4970383807230365
0.8326481279145556	0.1659321810188835	0.4970689017529312
0.8343661635982268	0.6646781555964774	0.4972146130512113
0.0027838565341527	0.1765064064620240	0.4406067667320310
0.0023831958113367	0.6763238922943356	0.4405807094749039
0.5026972085661721	0.1776627884122122	0.4407646334159433
0.5023484609174182	0.6703554342121980	0.4459071570829922
0.0023499842256132	0.3247779437237795	0.5534584279901013
0.0029649207559673	0.8248012071601243	0.5535052247031209
0.5015802101408416	0.3217234061326684	0.5512486264914273
0.5019867719203290	0.8256442798342527	0.5542319864402752

0.3281749293599002	0.3314087170372032	0.4455295678377801
0.3221621320427003	0.8255626494735061	0.4405120040206540
0.8219647629995904	0.3255105274839731	0.4406395760774122
0.8206630453849143	0.8238669000453624	0.4411173130648982
0.1727160654249068	0.1757471584525499	0.5540432054948051
0.1737102446631641	0.6774429845613088	0.5534603031882734
0.6740508223058846	0.1767290301585544	0.5535146124770509
0.6778434847426831	0.6809533308519720	0.5518724876323242
0.1744473810346544	0.4962583717401464	0.4408199280806809
0.1728538721744086	0.9959314305042666	0.4405004744341882
0.6671880518662945	0.4961067045996176	0.4459062136815183
0.6730203047494064	0.9955593643330745	0.4406713571340936
0.3188156545914944	0.4956610722612028	0.5512941042448762
0.3213631379557277	0.9960572389029987	0.5533406976108897
0.8225922004246298	0.4960265221846579	0.5542444259352564
0.8217555847381659	0.9967244782459712	0.5535605793449678
0.1645076747410398	0.3305999948680886	0.4971300529159828
0.1658901530636077	0.8326877548131788	0.4969839048262230
0.6681868339266533	0.3330277771924090	0.4973702760854530
0.6650613582381388	0.8341357704165456	0.4974993619961898
0.5092047328940164	0.5091603701338334	0.6259518187468961
0.4388946965056197	0.4374991607826342	0.6433213473823791

AlGaSi2S6-OOH

Generated by VASPKIT code

1.00000000000000

11.8949537277000008 0.0000000000000000 0.0000000000000000

-5.9474768639000004 10.3013321050000002 0.0000000000000000

0.0000000000000000 0.0000000000000000 27.7635459900000008

Si Ga S Al O H

8 4 24 4 2 1

Direct

0.9980988243553223 0.9995502515767486 0.5369417064492285

0.9985922449161859 0.5000665465500724 0.5369960244822536

0.4982904964245846 0.0000751787452611 0.5370714751168653

0.5003429392443967 0.5015603332245012 0.5625975335972558

0.9976053788798986 0.9991399718316184 0.4566338108759095

0.9990948654548191 0.4998350426845085 0.4566759068725874

0.4984753839667422 0.0007725201425530 0.4567308409974924

0.4986953299138423 0.4999386553241939 0.4707470043798493

0.3293132942601481 0.1650469598712856 0.4970223157913160

0.3321205301973003 0.6691717511840533 0.4966614563331345

0.8315757737327313 0.1666304661802762 0.4966946971926025

0.8341918847738878 0.6660346135012251 0.4967913285952429

0.0018003145389670 0.1772028465041733 0.4401792961003175

0.0018441530303838 0.6771936921301864 0.4401850754650689

0.5014279520922318	0.1780884844189143	0.4405520787581443
0.5017321844775209	0.6714952568756553	0.4451804925437804
0.0009360133336964	0.3253845800076318	0.5531675343888794
0.0025740200545566	0.8258425976535362	0.5530731817624142
0.5007613695567300	0.3215516914014794	0.5513145645166093
0.5012862901326279	0.8258519365940770	0.5537048353558911
0.3270817332562561	0.3316504216322457	0.4450961146871177
0.3212354009830151	0.8260873543623305	0.4401991885922754
0.8209570428629351	0.3260476407352856	0.4403157904132922
0.8202342110681154	0.8248187442417014	0.4404382595065613
0.1716551026588657	0.1764937995354200	0.5538535483782122
0.1725999781588725	0.6779323465970351	0.5531007696813076
0.6731869317166326	0.1770791937208083	0.5531663548304793
0.6788509126510777	0.6830345422740660	0.5513733547967682
0.1733491645444265	0.4966379306269648	0.4404244519158145
0.1725415337954137	0.9969918467895834	0.4402495609052425
0.6670886730389362	0.4969444133159115	0.4450912013648881
0.6722108560814501	0.9964870618273303	0.4402216100927055
0.3167674306742543	0.4956627896693745	0.5508677585704640
0.3204998707110627	0.9961882527496010	0.5532275181488555
0.8214682963581824	0.4968569353706144	0.5537896960679735
0.8212158827931370	0.9978483778266274	0.5530379329898156
0.1630684391740450	0.3307819855710460	0.4968617081686090

0.1653386167169382 0.8336627655759582 0.4966690053613392

0.6666836816964633 0.3335187354725655 0.4971054690931739

0.6650095399409780 0.8352926049542362 0.4969734255144052

0.5143736543763693 0.5196932694275146 0.6235942065396859

0.4024448539861374 0.4030178881251633 0.6447788488709111

0.3433790094198810 0.4368377831966854 0.6506433459352865

AlGaSi2Se6-O

1.00000000000000

12.5886259078999991 0.0000000000000000 0.0000000000000000

-6.2943129538999996 10.9020698350000007 0.0000000000000000

0.0000000000000000 0.0000000000000000 28.7129516602000017

Si Ga Se Al O

8 4 24 4 1

Direct

0.9962910341156028 0.9960134467076386 0.5368210326526555

0.0039865532923620 0.5002775874079642 0.5368210326526555

0.4997224125920352 0.0037089658843975 0.5368210326526555

0.5000000000000000 0.5000000000000000 0.5922020170285096

0.9943842678142514 0.9956606451518236 0.4580155617582790

0.0043393548481774 0.4987236226624293 0.4580155617582790

0.5012763773375712 0.0056157321857481 0.4580155617582790

0.5000000000000000 0.5000000000000000 0.4886684836648555

0.3214932048891357	0.1617042927968814	0.4933923893797215
0.3402111049077506	0.6785068101108692	0.4933923893797215
0.8333333440000033	0.1666666710000015	0.4991101402518940
0.8382957222031234	0.6597889100922542	0.4933923893797215
0.9987365503050576	0.1749134336042111	0.4394527638374894
0.0080500486451923	0.6769808153455821	0.4378828390183576
0.5037076328707017	0.1842573881691086	0.4399795562216788
0.5014527290307060	0.6653836644047552	0.4447359848763171
0.0062252423119033	0.3238409490743057	0.5551755321485812
0.9985237342797271	0.8187801093422310	0.5538234880738030
0.5014898761916178	0.3428309390512508	0.5462470132208010
0.5010265216347213	0.8269929844133811	0.5557041408613499
0.3346163355952452	0.3360690656259546	0.4447359848763171
0.3230191846544181	0.8310692342996134	0.4378828390183576
0.8250865663957888	0.3238231177008507	0.4394527638374894
0.8157426118308918	0.8194502457015961	0.4399795562216788
0.1730070155866188	0.1740335382213434	0.5557041408613499
0.1812198906577689	0.6797436259374995	0.5538234880738030
0.6761590509256951	0.1823842942376014	0.5551755321485812
0.6571690609487496	0.6586589381403712	0.5462470132208010
0.1805497542984033	0.4962923671292984	0.4399795562216788
0.1689307657003861	0.9919499513548082	0.4378828390183576
0.6639309343740452	0.4985472709692941	0.4447359848763171

0.6761768822991491 0.0012634496949412 0.4394527638374894

0.3413410618596294 0.4985101238083819 0.5462470132208010

0.3202563740625005 0.0014762657202727 0.5538234880738030

0.8259664617786567 0.4989734783652790 0.5557041408613499

0.8176157057623983 0.9937747576880961 0.5551755321485812

0.1649105645646629 0.3243163071800700 0.4960423841303477

0.1666666710000015 0.8333333440000033 0.4974948769458067

0.6756837078199350 0.3405942743845978 0.4960423841303477

0.6594057406154065 0.8350894504353414 0.4960423841303477

0.5000000000000000 0.5000000000000000 0.6457067035707850

AlGaSi2Se6-OH

1.00000000000000

12.5886259078999991 0.0000000000000000 0.0000000000000000

-6.2943129538999996 10.9020698350000007 0.0000000000000000

0.0000000000000000 0.0000000000000000 28.7129516602000017

Si Ga Se Al O H

8 4 24 4 1 1

Direct

0.9989325217207148 0.9987401627759180 0.5367632869556139

0.9998337648615176 0.4997538920079575 0.5366452811394262

0.4992518573102889 0.9996140729269191 0.5366258938205499

0.5015285752714174 0.5015625665054795 0.5664798571258608

0.9983857848778767	0.9984984045342971	0.4580254608231907
0.0001506304731241	0.4995020380059080	0.4579239578976935
0.4995609542499646	0.0002048742253074	0.4579001209852092
0.4990285292688184	0.4991060153453412	0.4754237137251454
0.3301237793895066	0.1648757601261235	0.4966845384976277
0.3338355123874904	0.6693146755224336	0.4970026237209463
0.8329084630388417	0.1660953892224623	0.4970171983862687
0.8339930084104541	0.6643923607319391	0.4973860946685272
0.0024633890012425	0.1776462435385695	0.4392445455832487
0.0016287467578767	0.6773326526283796	0.4391683300486321
0.5024202128444738	0.1788629794333244	0.4393725856677841
0.5022863228510497	0.6705816843769703	0.4447938364653894
0.0018740005498489	0.3237656941359172	0.5549469364286204
0.0028988798020575	0.8235595067362464	0.5548911116020372
0.5011079061000983	0.3183788372880674	0.5535775920837319
0.5014764214320512	0.8240676512079489	0.5559530567994040
0.3282906169184374	0.3312195677141082	0.4442512739448408
0.3213785140486518	0.8242070552461427	0.4390323668288353
0.8211575042265359	0.3241405252819423	0.4392629752568427
0.8196757712516869	0.8224091898726336	0.4397801167585158
0.1743954577251749	0.1769630747343640	0.5556690019698481
0.1751886905080593	0.6787363813313373	0.5547996510180341
0.6757477610838375	0.1772952851919985	0.5549636643901894

0.6818217691687326 0.6844852573510657 0.5545122957005184

0.1761102328618823 0.4968993130518579 0.4394311941005758

0.1745776325252409 0.9968085264373416 0.4390575894108367

0.6677087364848769 0.4966647883652824 0.4448122378190595

0.6746015300211977 0.9959835142129000 0.4393240948257329

0.3160168586654253 0.4960636451607180 0.5536310042822591

0.3206845426663397 0.9967718837601112 0.5547167415159513

0.8215313154828170 0.4964826271619090 0.5559920963964727

0.8213991005140338 0.9979034388516624 0.5550448507336062

0.1646906335485254 0.3306181310422189 0.4972206549611062

0.1660431952510692 0.8329229966253519 0.4968388789331424

0.6685161395641065 0.3331590453362183 0.4974690596194306

0.6651361051852712 0.8342030184884511 0.4977389033588498

0.5092702188607999 0.5091539414438544 0.6245575058599170

0.4423684648385741 0.4410534000630685 0.6410680998905156

AlGaSi2Se6-OOH

1.00000000000000

12.5886259078999991 0.0000000000000000 0.0000000000000000

-6.2943129538999996 10.9020698350000007 0.0000000000000000

0.0000000000000000 0.0000000000000000 28.7129516602000017

Si Ga Se Al O H

8 4 24 4 2 1

Direct

0.0012330651718663	0.9990476474299894	0.5365679101925824
0.0014735280425828	0.4997834793769213	0.5367556933249974
0.5012571358115123	0.9992613072252275	0.5367247731745453
0.5036177882626555	0.5012703474528042	0.5625769967409963
0.0007742759765624	0.9986835341311503	0.4578566248202270
0.0020358230206327	0.4994479022683122	0.4580525071876021
0.5014172400043709	0.0000430653289254	0.4580072514488190
0.5018893573327224	0.4997093996857150	0.4738116117181304
0.3325686516130432	0.1652313708067134	0.4976959134951951
0.3352917188613288	0.6686108909474531	0.4972848811164507
0.8347255182683931	0.1661938949084179	0.4970330712877183
0.8366894492615682	0.6648842883417742	0.4971385556752386
0.0045870498242796	0.1778793876248423	0.4393046176481585
0.0041586191207110	0.6775980155857637	0.4391675350335573
0.5038807310866631	0.1785464696736092	0.4396195517042106
0.5042115023233807	0.6711582691518104	0.4443670168593953
0.0031864295069842	0.3237382704244094	0.5552306809593258
0.0051315102818022	0.8238746012293778	0.5548237969125359
0.5031572581770501	0.3175584268896875	0.5542418437952695
0.5038349939999583	0.8236681296838488	0.5558275444267803
0.3299059167848548	0.3306949807912707	0.4445096351111550
0.3232477226034925	0.8239042266661589	0.4392523137130101

0.8229731178325979	0.3241062880534708	0.4394443483137895
0.8222443307128453	0.8228003055728964	0.4393482317939252
0.1760173140536886	0.1767872478598468	0.5561884946901666
0.1767847315707400	0.6787296379471840	0.5549912689659489
0.6777337725046331	0.1769083309197530	0.5550777019445107
0.6857401321078128	0.6858484101212446	0.5540590158732668
0.1778551274719937	0.4964891605199436	0.4396366051277735
0.1774470967207228	0.9975212015991862	0.4393398600100505
0.6704380829554285	0.4965445324348243	0.4442230943047989
0.6766546428361334	0.9963721764223017	0.4391992606096976
0.3167831186269824	0.4953758862242255	0.5538082515936854
0.3227791115662560	0.9961275402573344	0.5551567747369260
0.8232333253009475	0.4967878419041770	0.5559504105316138
0.8236395314601933	0.9983513597388564	0.5548071521355078
0.1660387102481139	0.3301024571468401	0.4976708199240176
0.1683644759311631	0.8333533753935962	0.4970021227828251
0.6695331702948125	0.3328905643266645	0.4977451788381527
0.6679493435616398	0.8347556989148691	0.4974247549971443
0.5200613239868178	0.5207004083687142	0.6218401439273493
0.4165632094883071	0.4124374344074613	0.6446635294822552
0.3628910974317458	0.4462223042424689	0.6515729330706844

References

[1] J. K. Norskov, T. Bligaard, A. Logadottir, J. Kitchin, J. G. Chen, S. Pandelov, J. Norskov, Trends in the exchange current for hydrogen evolution, J. Electrochem. Soc. 152 (3) (2005) J23–J26.

[2] C. Quan, S. Ji, R. Yao, W. Liu, J. Yang, X. Li, Two-dimensional janus AgBiP2X3X3′ (X, X′ = S, Se, Te): Efficient intrinsic electric field regulatory strategy for photocatalytic overall water-splitting, Int. J. Hydrog. Energy 56 (2024) 1227–1234.

[3] T. V. Vu, N. N. Hieu, D. D. Vo, A. Kartamyshev, H. D. Tong, T. T. Trinh, V. Khuong Dien, Z. Haman, P. Dey, N. Khossossi, 2D Ge2Se2P4 monolayer: A versatile photocatalyst for sustainable water splitting, J. Phys. Chem. C 128 (10) (2024) 4245–4257.

[4] E. Skúlason, T. Bligaard, S. Gudmundsdóttir, F. Studt, J. Rossmeisl, F. Abild-Pedersen, T. Vegge, H. Jónsson, J.K. Nørskov, A theoretical evaluation of possible transition metal electro-catalysts for N2 reduction, Phys. Chem. Chem. Phys., 14 (2012), 1235-1245.

[5] P. D. Trung, H. D. Tong, First-principles study of Ga2Ge2S3Se3 monolayer: a promising photocatalyst for water splitting, RSC Adv. 15 (2025) 8060–8071.

[6] C.-F. Fu, J. Sun, Q. Luo, X. Li, W. Hu, J. Yang, Intrinsic electric fields in two-dimensional materials boost the solar-to-hydrogen efficiency for photocatalytic water splitting, Nano Lett. 18 (10) (2018) 6312–6317.
